# Advanced Extraction Techniques Combined with Natural Deep Eutectic Solvents for Extracting Phenolic Compounds from Pomegranate (*Punica granatum* L.) Peels

**DOI:** 10.3390/ijms25189992

**Published:** 2024-09-17

**Authors:** Isadora Lopes de Oliveira, Gloria Domínguez-Rodríguez, Lidia Montero, Juliane Viganó, Alejandro Cifuentes, Mauricio Arial Rostagno, Elena Ibáñez

**Affiliations:** 1 Multidisciplinary Laboratory of Food and Health (LabMAS), School of Applied Sciences (FCA), Universidade Estadual de Campinas, São Paulo 13083-871, Brazil; isadoralopesdeoliveira1@gmail.com; 2 Institute of Food Science Research (CIAL-CSIC), Nicolás Cabrera 9, Campus UAM Cantoblanco, 28049 Madrid, Spain; lidia.montero@csic.es (L.M.); a.cifuentes@csic.es (A.C.); elena.ibanez@csic.es (E.I.); 3 Departamento de Química Analítica, Química Física e Ingeniería Química, Facultad de Ciencias, Universidad de Alcalá, Ctra. Madrid-Barcelona Km. 33.600, Alcalá de Henares, 28871 Madrid, Spain; 4 Department of Food Engineering, Faculty of Animal Science and Food Engineering, University of São Paulo, Av. Duque de Caxias Norte, 225, Pirassununga 13635-900, Brazil; julianevigano@gmail.com

**Keywords:** ellagic acid, α-punicalagin, β-punicalagin, natural deep eutectic solvents, ultrasonic-assisted extraction, pressurized liquid extraction

## Abstract

Pomegranate (*Punica granatum* L.) peel is a potential source of bioactive phenolic compounds such as ellagic acid and α- and β-punicalagin. This work explores the efficiency of natural deep eutectic solvents combined with ultrasound-assisted extraction (UAE) and pressurized liquid extraction (PLE) for their extraction. Five NaDESs were evaluated by employing UAE (25 °C, for 50 min) to determine their total phenolic content (Folin–Ciocalteu assay) and ellagic acid and α- and β-punicalagin contents (high-performance liquid chromatography (HPLC-DAD)). The NaDES composed of choline chloride (ChCl) and glycerol (Gly) (1:2, molar ratio) was the most efficient in the UAE when compared with the rest of the NaDESs and water extracts. Therefore, ChCl:Gly was further evaluated using PLE at different temperatures (40, 80, 120 and 160 °C). The PLE-NaDES extract obtained at 80 °C for 20 min at 1500 psi exhibited the highest contents of ellagic acid and α- and β-punicalagin compared to the rest of the temperatures and PLE-water extracts obtained under the same extraction conditions. Combining UAE or PLE with a NaDES emerges as a sustainable alternative for extracting ellagic acid and α- and β-punicalagin from pomegranate peel.

## 1. Introduction

The food industry generates around 37 million tons of waste annually worldwide. In particular, the fruit and vegetable industry generates the most waste (29.3%) compared to other foods such as cereals (7.8%), meat (5.5%), and dairy products (10.3%) [1]. The high volume of waste has adverse effects on the environment and human health due to microbial decomposition and leachate production, which are caused by its poor biological stability, high concentration of organic compounds, high water activity, and poor oxidative stability [2,3].

This waste is generally burned to remove fungi and parasites, which entails a great economic expense for the companies involved. However, several studies have shown that fruit by-products are a source of interesting bioactive compounds, which could be used in the pharmaceutical, cosmetic, and food industries. Thus, several researchers are focusing on transitioning toward a circular economy based on resource efficiency and eco-innovation principles, centered on revalorizing waste from the food industry for commercial purposes [4].

Pomegranate (*Punica granatum* L.) is widely consumed in fresh and processed forms, such as juices or jams. It is estimated that global production reaches approximately 3–4 million metric tons annually [5]. From this total production, 50–65% becomes by-products during processing, mainly constituting seeds and peels, representing up to 23% and 73% of pomegranate weight, respectively [6]. Pomegranate peels and seeds are characterized by interesting biological properties, such as antioxidant, antidiabetic, and antibacterial properties [7]. In general, the bioactive properties of pomegranate peel have been attributed to its content of phenolic compounds such as phenolic acids, flavonoids, and tannins [7,8,9,10]. Among the phenolic compounds, ellagic acid and α- and β-punicalagin have been identified as the main phenolic compounds found in pomegranate peel [11]. These phenolic compounds have shown interesting antioxidant, anti-cancer, antigenotoxic, and neuroprotective capacities, among others [11,12].

Typically, these phenolic compounds have been extracted from pomegranate by-products using traditional extraction methods that utilize water and organic solvents, such as ethanol, methanol, or their combinations [12,13,14,15]. Nevertheless, conventional extraction methods are time-consuming and demand a substantial volume of solvents. To avoid these negative aspects, advanced extraction techniques such as ultrasound-assisted extraction (UAE) and pressurized liquid extraction (PLE) have appeared as more environmentally friendly alternatives to reduce the amount of solvent and extraction time [16,17].

In addition, in the last few years, a new generation of solvents called natural deep eutectic solvents (NaDESs) has emerged as an alternative to organic solvents to extract bioactive compounds. NaDESs consist of a mixture of two or more organic components, which are primary metabolites from living organisms. NaDESs have melting points lower than each individual component. Furthermore, due to the nature of the NaDES components, these solvents have been declared as cheaper, non-toxic, biodegradable, and easy-to-obtain compared to conventional organic solvents [18]. NaDESs are formed by hydrogen bonds, as well as van der Waals interactions between a hydrogen bond acceptor (HBA) and a hydrogen bond donor (HBD). Usually, choline chloride quaternary salt is used as the HBA, while amino acids, sugars, or carboxylic acids such as glucose, glycerol, lactic acid, or acetic acid are used as the HBD for the extraction of phenolic compounds. Therefore, there are many combinations of different components that can be adjusted to form NaDESs, and the most suitable mixture may be pursued for a specific application. However, different parameters must be considered to synthesize NaDESs, in order to obtain the viscosity, conductivity, density, and polarity suitable for extracting the target compounds [18,19]. 

In this manner, NaDESs have been employed for the extraction of bioactive compounds by maceration but also in combination with advanced extraction techniques such as UAE or PLE [7,20,21]. Bertolo et al. [8] observed a high efficiency in the extraction of phenolic compounds from pomegranate peels using UAE combined with a NaDES composed of choline chloride with 35% lactic acid and 20% water compared to ethanolic extracts. Additionally, choline chloride combined with propylene glycol, malic acid, citric acid, or oxalic acid has also been used for the recovery of phenolic compounds from different food matrices, such as soy and berry by-products and grape pomace [22,23,24,25]. Nevertheless, NaDESs have scarcely been used to extract phenolic compounds from pomegranate peels. In fact, to our knowledge, there are no studies on the combination of PLE with a NaDES for the extraction of specific phenolic compounds such as ellagic acid and α- and β-punicalagin from pomegranate peels.

Therefore, this study investigated the efficiency of NaDESs in the recovery of ellagic acid and α- and β-punicalagin from pomegranate peels based on its combination with UAE and PLE in comparison with water as the solvent used for extraction. First, the physicochemical characteristics of different NaDESs were evaluated by considering their pH, viscosity, and density at different temperatures. Five NaDESs derived from choline chloride were tested to determine the best to obtain the highest content of target compounds from pomegranate peels when employing UAE-NaDES. With the selected NaDESs, the influence of the extraction temperature in the recovery of the target compounds was evaluated by using 40, 80, 120, and 160 °C in PLE-NaDES extraction. UAE-NaDES and PLE-NaDES extracts were compared with extracts obtained under the same extraction conditions but using water instead of a NaDES as the extraction solvent in terms of the total phenolic content determined by a Folin–Ciocalteu assay and the ellagic acid and α-punicalagin and β-punicalagin contents analyzed by liquid chromatography coupled with diode array detection (HPLC-DAD). Figure S1 shows the procedure carried out in this study for optimizing the ellagic acid and α- and β-punicalagin extraction process. 

## 2. Results

### 2.1. Physicochemical Characterization of NaDESs

The recovery of phenolic compounds from natural matrices depends on the physicochemical properties of the solvent used for this purpose. Thus, different NaDESs were selected according to the literature to extract phenolic compounds from food matrices (Table 1); these were tested in the extraction of phenolic compounds from pomegranate peel and, in particular, for their capacity to obtain high contents of ellagic acid and α- and β-punicalagin.

These NaDESs were synthesized using 40% water to increase the phenolic compound extraction efficiency, as according to Vo et al. [24]. The physicochemical properties of the synthesized NaDESs (pH, viscosity, and density) were evaluated, and the results are shown in Table 2.

As can be seen in Table 2, regardless of the temperature, the pH was different depending on the HBD component. In this sense, the NaDESs composed of ChCl:LA (1:1 and 1:2) presented the lowest pH, while the NaDES composed of ChCl:Glu (1:2) showed the highest pH values.

Viscosity is another physicochemical property that should be investigated since it is also involved in solvent toxicity and extraction efficiency. The NaDES viscosity depends on the nature of each component, the percentage of water added in its synthesis, as well as the temperature applied to the solvent. Thus, the viscosity of NaDESs was tested in this study at different temperatures up to the boiling point of water. As can be observed in Table 2, a substantial reduction in the viscosity with the increasing temperature was observed. In fact, the viscosity of the NaDES composed of ChCh:LA (1:1), which presented the highest viscosity at 25 °C, was reduced by almost six times when a temperature of 100 °C was applied. All NaDESs showed viscosity values below 9 mPas·s at 100 °C. Notably, the NaDES composed of ChCl:AA (1:1) presented the lowest viscosity, while ChCl:LA (1:1) showed the highest viscosity at 25 °C. However, when the temperature was increased, similar viscosity values were observed among all NaDESs, though we highlight ChCl:LA (1:1) and ChCl:Glu as having the highest viscosity values during the temperature increase.

Similar to viscosity, the increase in temperature slightly decreased the density of the ChCl:AA (1:1) and ChCl:LA (1:2) NaDESs, as is shown in Table 2.

Following the same trend as viscosity, the NaDES composed of ChCl:Glu (1:2) presented the highest density, while the NaDESs composed of ChCl:AA (1:1) and ChCl:Gly (1:2) showed the lowest values. Together with viscosity, this is an important parameter to consider when designing the extraction process.

### 2.2. NaDES Screening Applying UAE

To select the best NaDES for extracting phenolic compounds from pomegranate peels, the five different NaDESs were tested by applying UAE. Based on our previous experience of optimizing the UAE-NaDES extraction conditions to obtain phenolic compounds from food by-products, the extraction conditions reported in Section 4.5 and according to Cvjetko et al.’s [25] protocol were applied. The same extraction conditions were used for each NaDES in order to determine the best for the recovery of the highest TPC, as evaluated by the Folin–Ciocalteu assay, and the highest contents of ellagic acid and α- and β-punicalagin, as measured by HPLC-DAD. In addition, UAE-NaDES extracts were compared with UAE-water extracts obtained under the same extraction conditions to determine the efficiency of NaDESs in extraction.

**Figure 1:**
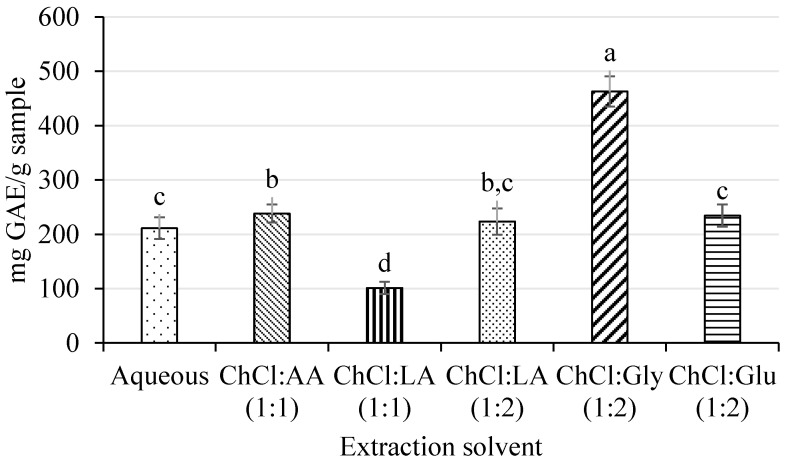
Total phenolic content (TPC) determined by the Folin–Ciocalteu assay of UAE-NaDES and UAE-water extracts, expressed as mg GAE/g sample. ^a–d^ Letters indicate statistically significant differences (*p* ≤ 0.05) among NaDESs.

All extracts were compared in terms of TPC, and the results are shown in Figure 1. The extract obtained using ChCl:Gly (1:2) presented the highest TPC values compared to the rest of UAE-NaDES extracts. In fact, ChCl:Gly (1:2) produced more than twice the TPC values than the extract obtained using water as the extraction solvent. This means that this NaDES was more effective in extracting phenolic compounds than conventional solvents.

Although the Folin–Ciocalteu method is widely employed for providing an approximate quantification of the total phenolic contents of the extracts, this method can produce an overestimation in the phenolic quantification, due to the ability of other compounds present in the sample to donate electrons and participate in the redox reaction. Thus, more accurate methods for quantifying phenolic compounds are necessary, such as HPLC-DAD. 

In this sense, UAE-NaDES extracts were analyzed by HPLC-DAD for an exhaustive identification and quantification of their main compounds, ellagic acid and α- and β punicalagin, using their respective standards. Table 3 shows a comparison of ellagic acid and α- and β punicalagin recovery by HPLC-DAD from the different studied NaDESs. 

As can be observed in Table 3, ChCl:Gly (1:2) and ChCl:LA (1:1) were the most efficient NaDESs for the recovery of the three targeted phenolic compounds. This agrees with the physicochemical parameters of NaDESs determined in this work, where it was observed that NaDESs with low viscosities and acidic pH values increased the extraction of phenolic compounds and, particularly, ellagic acid and α- and β-punicalagins from pomegranate peels. In particular, the NaDES composed of ChCl:Gly (1:2) allowed for extracting the highest ellagic acid and α-punicalagin contents with statistically significant differences from the rest of the NaDESs. In addition, despite the water extract presenting a high content of α-punicalagin without significant differences compared to the ChCl:Gly extract, water did not allow for an efficient recovery of ellagic acid and β-punicalagin from pomegranate peels compared to this NaDES.

Despite the combination of advanced extraction techniques with NaDESs presenting a promising tool for the recovery of phenolic compounds, studies on the extraction of ellagic acid and α- and β-punicalagin using this combination are very limited. As a preliminary study, these results indicate that the use of NaDESs under specific extraction conditions improves the extraction of phenolic compounds, particularly ellagic acid and α- and β-punicalagins, from pomegranate peels when compared with conventional solvents. In addition, this study proposes that ChCl:Gly (1:2) is the most efficient NaDES combined with UAE, which could serve as the starting point for subsequent optimization of UAE extraction parameters and improve the extraction. 

On the other hand, there are no studies on the use of PLE combined with NaDESs for the recovery of ellagic acid and α- and β-punicalagin from pomegranate peels. Thus, considering the good results obtained by combining UAE with ChCl:Gly (1:2) NaDES in addition to the high efficiency of this NaDES in the extraction of phenolic compounds from several matrices [26,27,28], PLE extraction of the pomegranate peel was also evaluated, using the same extraction solvent, as another emergent and green alternative for the valorization of food by-products.

### 2.3. Effect of PLE Temperature

Once ChCl:Gly (1:2) was selected for the extraction of ellagic acid and α- and β-punicalagin from pomegranate peels, this NaDES was studied for PLE-NaDES extraction. Based on our previous experience in extracting phenolic compounds by using PLE from several food matrices, we considered that the nature of the extracting solvent and the extraction temperature were the most influential parameters on the extraction efficiency. Accordingly, the extraction method employed by Sánchez-Martínez et al. [29] was applied, with us setting the extraction time at 20 min and the pressure at 10.5 MPa. In addition, taking into account that degradation of target compounds occurs at high temperatures, this parameter was optimized. Temperatures of 40, 80, 120, and 160 °C were tested determining the TPC by using the Folin–Ciocalteu assay and the ellagic acid and α- and β-punicalagin contents by using HPLC-DAD; these were our response variables. 

**Figure 2:**
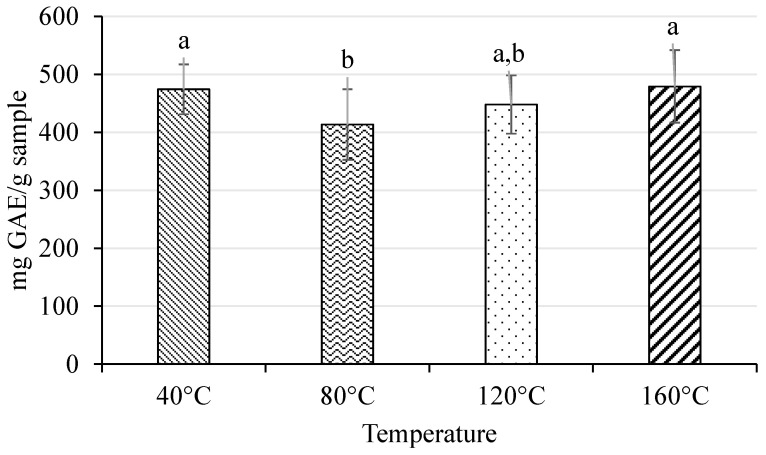
Total phenolic content (TPC) expressed as mg GAE/g sample of PLE-NaDES (ChCl:Gly (1:2)) extracts obtained at different temperatures (40, 80, 120, and 160 °C). ^a,b^ Letters indicate statistically significant differences (*p* ≤ 0.05) among extraction temperatures.

As can be observed in Figure 2, extraction temperatures of 80 °C and 120 °C resulted in extracts with statistically significantly lower TPC values (413 ± 61 and 448 ± 50 mg GAE/g sample, respectively) compared to the use of 40 °C. On the other hand, extracts obtained at 40 °C and 160 °C presented the highest TPC values, without statistically significant differences between them.

**Figure 3:**
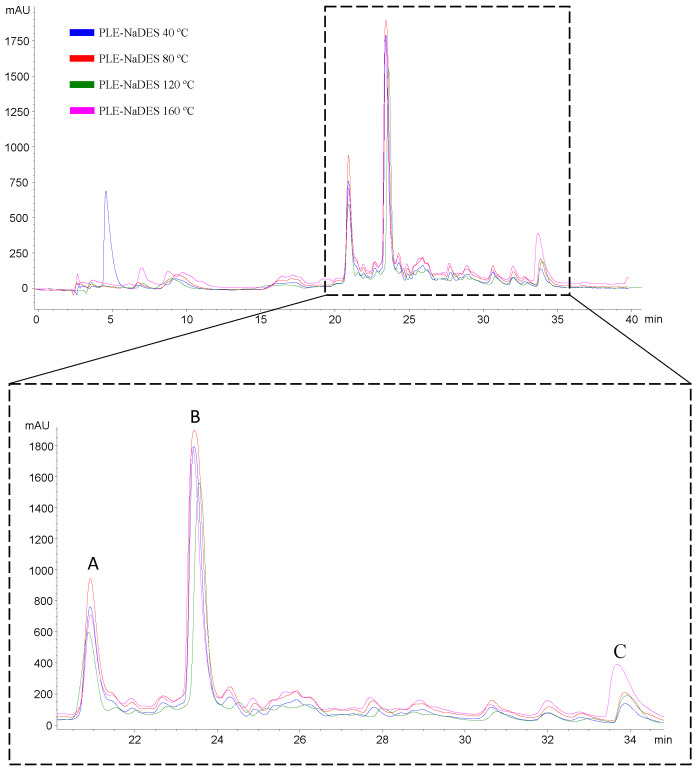
LC-DAD chromatogram (280 nm) of the PLE-NaDES pomegranate peel extracts at different temperatures using ChCl:Gly (1:2) as the extracting solvent: (A) α-punicalagin, (B) β-punicalagin, (C) ellagic acid.

However, although the TPC assay showed a higher phenolic content at 40 °C and a reduction in this value at 80 °C, the results of the specific quantification of ellagic acid and α- and β-punicalagin using HPLC-DAD from PLE-NaDES extracts (Table 4 and Figure 3) showed that PLE extraction temperature of 80 °C did not produce a degradation of ellagic acid and α- and β-punicalagin; instead, this temperature provided higher concentrations of α- and β-punicalagin (93 and 114 µg/g sample, respectively).

These compounds seemed to resist extraction temperatures of up to 80 °C, while other phenolic compounds were degraded, resulting in lower TPC values. HPLC-DAD analysis revealed an increase in the ellagic acid content with the increase in extraction temperature. In contrast, a decrease in α- and β-punicalagin contents was observed with the increase in extraction temperature from 80 °C (Figure 3 and Table 4).

On the other hand, to evaluate the effectiveness of NaDESs in the extraction of ellagic acid and α- and β-punicalagin by PLE from pomegranate peel, one extraction at 80 °C (optimized PLE temperature) was performed using water instead of a NaDES. In this manner, ellagic acid and α- and β-punicalagin extraction was compared under PLE conditions with two different solvents (NaDES and water). Table 4 shows that the extraction of ellagic acid using water instead of a NaDES was statistically significantly reduced from 216 to 117 µg/g sample, while the concentration of β-punicalagin also presented a statistically significant difference, being reduced from 114 to 84 µg/g sample. Meanwhile, no statistically significant differences were observed in the α-punicalagin content (93 and 94 µg/g sample for NaDES and water extraction, respectively). Thus, the NaDES was more efficient in the extraction of these phenolic compounds than water. We propose that the physicochemical properties of the NaDES with 40% water increased the mass transfer rate and the penetration of the solvent into the matrix, improving the extraction of phenolic compounds from pomegranate peels compared to 100% water.

### 2.4. Influence of the Advanced Extraction Technique Used on the Recovery of Target Compounds

**Figure 4:**
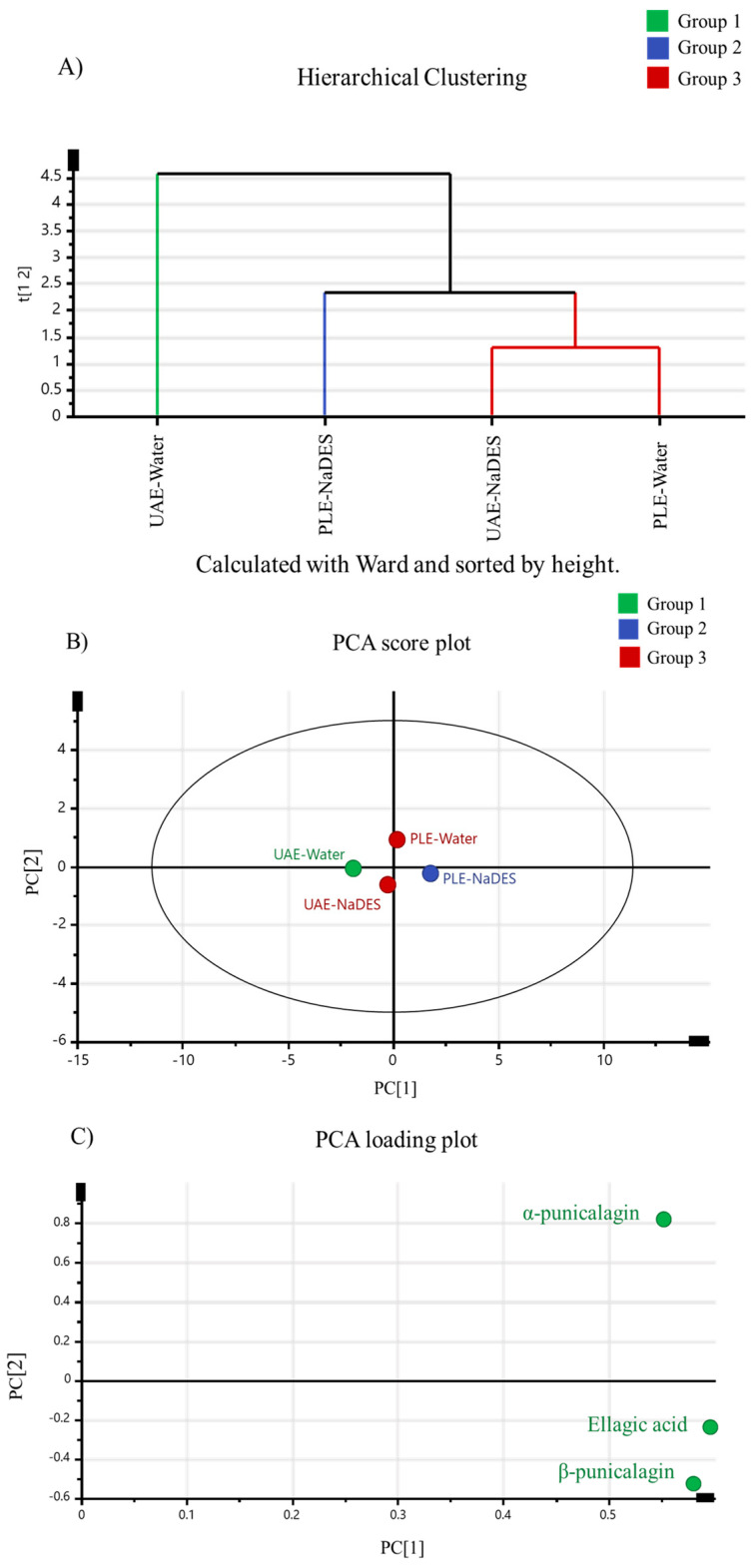
(**A**) Dendrogram achieved by HCA employing the Ward method and shortened to the heights of ellagic acid and α and β-punicalagin from UAE-NaDES, PLE-NaDES, UAE-water, and PLE-water extracts obtained under the optimal extraction conditions from pomegranate peel. (**B**) Score plot from PCA of different extractions from pomegranate peel. (**C**) Loading plot obtained from PCA.

A multivariate statistical analysis was performed to determine the influence of the advanced extraction technique in combination with a NaDES on the extraction of ellagic acid and α- and β-punicalagin, as well as the effectiveness of a NaDES instead of water as the extraction solvent (Figure 4). For that, optimized UAE-NaDES (using ChCl:Gly at 25 °C) and PLE-NaDES (using ChCl:Gly at 80 °C) were compared with extracts obtained under their respective extraction conditions using water instead of a NaDES as the extraction solvent (UAE-water and PLE-water extracts, respectively).

Pomegranate peel extracts were divided into groups according to their ellagic acid and α- and β-punicalagin contents determined by HPLC-DAD, obtaining three clusters (Figure 4A) in a hierarchical cluster analysis (HCA). One cluster (colored in green) grouped one extract (UAE-water), and the second cluster grouped another extract (PLE-water), colored in blue. In contrast, the two other extracts (UAE-NaDES and PLE-NaDES) were categorized in a third cluster (colored in red). The separation of the extracts according to their phenolic composition was carried out by PCA, which selected the most significant variables (principal components). Two principal components described 91% of the total data variability, grouping the extracts similarly to HCA (using the same group colors for HCA and PCA) (Figure 4A,B).

Extracts belonging to the same group showed similar ellagic acid and α- and β-punicalagin contents. In this sense, when overlapping Figure 4B with Figure 4C, it can be observed that group 1 (corresponding to the UAE-water extract) had lower ellagic acid and α- and β-punicalagin contents, which were in the opposite site to the phenolic compounds in the PCA loading plot. This extract presented a lower phenolic content than the UAE-NaDES extract (group 3) obtained under the same extraction conditions. The PLE-water extract obtained with a higher extraction temperature and shorter extraction time (80 °C for 20 min) than the UAE-water and UAE-NaDES extracts (25 °C for 50 min) was more effective in the extraction of phenolic compounds than UAE-water but similar to the UAE-NaDES extract. This effect can be observed in Figure 4B, where the PLE-water extract is in the third group along with the UAE-NaDES extract, but in a different group from the UAE-water extract. In particular, PLE-water allowed for obtaining a higher α-punicalagin content, while UAE-NaDES achieved higher ellagic acid and β-punicalagin contents according to the PCA loading plot. Notably, group 2, which corresponded to the PLE-NaDES extract, presented the highest phenolic contents compared to the rest of the extracts, with the highest ellagic acid and β-punicalagin contents in the same site in the PCA loading plot. The differentiation in groups between the PLE-water and PLE-NaDES extracts corroborated that the use of a NaDES in PLE increases the extraction of target compounds when compared with conventional solvents such as water, under the same extraction conditions. In general, PLE-NaDES extraction at 80 °C was the most effective process for extracting ellagic acid and α- and β-punicalagin from pomegranate peels; it was more effective compared to PLE-water extraction at 80 °C, and compared to UAE-NaDES and UAE-water extraction at 25 °C.

## 3. Discussion

NaDESs are based on the mixture of two or more components (HBA and HBD components) that confer to the solvent a different pH, viscosity, and density, influencing the extraction efficiency of bioactive compounds. In this manner, components of NaDESs can be selected according to their physicochemical properties to enhance the solubility of target compounds [19,30]. 

The pH is an important physicochemical parameter of NaDESs since it has a different influence on the extractability and toxicity of the formed solvent. In this sense, it is known that the acidity of NaDESs increases the extraction of anthocyanins from food matrices once the acidic medium stabilizes the flavylium ion, enhancing their extraction [31,32,33]. In addition, an acidic NaDES produces cell wall breakdown with a greater delignification ability than a basic NaDES [31]. In this sense, the pH values of the NaDESs used in this study (Table 2) indicated that the NaDES composed of ChCl:LA (1:1 and 1:2) could provide the highest recovery of anthocyanins and the highest release of phenolic compounds from the cell wall from pomegranate peel due to having the lowest pH. On the other hand, the ChCl:Glu (1:2) NaDES showed the highest pH values, and this NaDES has been widely used for the extraction of flavonoids such as hesperidin or quercetin, among others [32,33]. Usually, alcohols or sugar-based systems are employed to extract this type of phenolic compounds [32,33]. In terms of toxicity, despite NaDESs being considered non-toxic solvents, different researchers have observed that the acidic conditions of a NaDES influence its cytotoxicity. For example, Radošević et al. [34] observed a low cytotoxic effect of ChCl:Glu and ChCl:Gly NaDESs on fish and human cell lines, while these cell lines experienced moderate cytotoxicity when using ChCl:oxalic acid [34]. 

However, the extraction efficiency of a NaDES not only depends on its pH but is also highly dependent on viscosity. The high viscosity of NaDESs is attributed to hydrogen bonds and van der Walls and electrostatic interactions determined by the molar ratio and the type and number of NaDES components that form the eutectic mixture [35]. This high viscosity also negatively affects the extraction efficiency of bioactive compounds, and it can be resolved by adding water to NaDESs. Even if this is a common practice, it should be considered that a water content in a NaDES higher than 60% produces a destabilization of hydrogen bonds of the NaDES, giving rise to an aqueous solution where NaDES components are dissolved [36]. Thus, 40% water was added to the different NaDESs tested in this study, according to Vo et al.’s investigation of how to obtain phenolic compounds from mulberry fruits [24]. In addition, the use of high temperatures during extraction is recommended to reduce the viscosity of the solvent, enhancing its penetration into the matrix for the recovery of bioactive compounds [37,38]. That viscosity reduction when the temperature is increased was demonstrated in this study (Table 2). Taking into account the results obtained for the physicochemical properties of NaDESs, it seems the NaDES composed of ChCl:AA (1:1) could have a better penetration into the matrix for the recovery of phenolic compounds due to its low viscosity and density, along with ChCl:Gly (1:2) and ChCl:LA (1:2), compared to the rest of the NaDESs when temperature is applied. 

In particular, when the extraction of phenolic compounds from pomegranate by UAE-NaDES was carried out at 25 °C for 50 min, the NaDES composed of ChCl:Gly (1:2) provided UAE extracts with the highest TPC values and the highest ellagic acid and, α- and β-punicalagin contents when compared with the rest of the NaDESs (Figure 1 and Table 3). In the literature, a NaDES composed of ChCl:Gly with water has shown a high efficiency in the extraction of phenolic compounds from different food matrices, not only because of the lower viscosity shown but also due to its high polarity and dielectric constraint [26,27], and the strong interaction of ChCl and Gly with phenolic compounds, which facilitates its extraction [28]. Additionally, this NaDES is also used for its pH value below 2, which can help in hydrolyzing the cell wall of fruit peel, facilitating the extraction of phenolic compounds [39]. In this study, lower pH values were preferred for the extraction of phenolic compounds but not lower than pH 1. In fact, extracts obtained with ChCl:AA and ChCl:Gly with pH values of 1.40 and 1.60, respectively, presented the highest TPC values compared to the rest of UAE-NaDES extracts, while those achieved in extreme pH conditions, such as those obtained using ChCl:LA (1:2) (pH 0.8), could degrade the phenolic compounds. For these reasons, ChCl:Gly (1:2) was selected as the best NaDES for extracting phenolic compounds from pomegranate peel. 

In addition, the efficiency of NaDESs in the recovery of phenolic compounds from pomegranate peel by UAE was demonstrated. Higher TPC values were obtained using UAE combined with a NaDES when compared with UAE employing water as the solvent. The efficiency of the combination of UAE with a NaDES has been demonstrated by several researchers, who revealed that the cavitation bubbles created by ultrasound waves destroy the cell walls, increasing the extraction yields, which are enhanced even more when using a NaDES as the extraction solvent [18,40,41]. In particular, Kim et al. [40] carried out a screening to select the NaDES to be used for obtaining punicalagin and ellagic acid from pomegranate peel, obtaining the highest contents when using ChCl:oxalic acid as the NaDES. However, this NaDES achieved lower punicalagin and ellagic contents than we did in the results obtained in this study.

On the other hand, a different form of NaDES behavior could occur when it is submitted to high pressures and temperatures. It is known that some thermolabile phenolic compounds can be degraded at high temperatures and, for this reason, the stability of target compounds must be considered for the extraction. In addition, the extraction temperature influences the NaDES viscosity and could be conclusive for its application in extraction systems since lower viscosities imply a lower internal resistance of the molecules, increasing their fluency, avoiding instrument obstructions, and helping the pumping process in the instrument [35]. Thus, to study the influence of pressure and temperature on the recovery of phenolic compounds from pomegranate peels, an optimization of PLE-NaDES extraction of ellagic acid and α- and β-punicalagin from these fruit peels was carried out. This study was performed using ChCl:Gly (1:2) NaDES as this solvent provided the best results in UAE in addition to achieving the best extraction efficiencies of phenolic compounds from several matrices [26,27,28,42]. Four different extraction temperatures were tested with this NaDES. Extraction temperatures higher than 100 °C imply the degradation of thermolabile phenolic compounds. In fact, Sumere et al. [38] and Chaves et al. [43] observed that extraction temperatures between 40 and 60 °C are the most suitable for extracting phenolic compounds from food matrices by PLE. An extraction temperature of 25 °C was tested in this study; however, an obstruction occurred in the PLE instrument due to the ChCl:Gly (1:2) NaDES viscosity. This indicated that higher PLE extraction temperatures are necessary to reduce the viscosity of NaDESs and avoid instrument obstruction. Thus, extraction temperatures from 40 °C to 160 °C were tested. In this study, a slight increase in TPC values was observed from an extraction temperature of 80 °C to 160 °C (see Figure 2). This could be related to the production of Maillard reaction products, which may lead to an overestimation of the phenolic content of pomegranate peel by interfering with the Folin–Ciocalteu method [44]. In fact, the extract obtained at 160 °C presented a dark color with a candy smell, characteristic of the Maillard reaction.

In addition, an increase in ellagic acid content was observed from 80 °C with respective reductions in α- and β-punicalagin contents (see Table 4 and Figure 3). This suggests that temperatures above 80 °C might produce the hydrolysis of punicalagins into ellagic acid. Punicalagins are ellagitannins that are highly bioavailable; once absorbed in our body, they are hydrolyzed, producing free ellagic acid and urolithins with a high antioxidant capacity [45]. However, the free form of ellagic acid is poorly bioavailable, and consequently, its ability to provide antioxidant activity is reduced [45]. Thus, it is essential to determine the content of free ellagic acid and punicalagins in food matrices to obtain a broad knowledge of their bioavailable ellagic acid content. In this sense, the higher extraction temperature used to determine the free ellagic acid and punicalagin contents was 80 °C since, at this temperature, a higher concentration of punicalagins was achieved, showing lower hydrolysis and transformation of these compounds in ellagic acid than at the higher tested temperatures. Thus, an extraction temperature of 80 °C was selected as the best extraction condition to obtain high contents of ellagic acid and α- and β-punicalagin via PLE-NaDES (ChCl:Gly, 1:2). In addition, the efficiency of a NaDES applied in PLE was demonstrated by comparison with a PLE-water extract at 80 °C. NaDES increase the recovery of bioactive compounds when using conventional extraction techniques such as maceration but also combined with advanced extraction techniques such as UAE and PLE.

## 4. Materials and Methods

### 4.1. Chemical and Reagents

HPLC-grade solvents, including acetonitrile and acetic acid (99.99%), were sourced from VWR Chemicals (Barcelona, Spain). Methanol (99.99%) was acquired from Fisher Scientific (Leicestershire, UK). Potassium persulfate, sodium carbonate, choline chloride, and gallic acid were obtained from Sigma-Aldrich (St. Louis, MO, USA). TCI Chemicals (Tokyo, Japan) provided Folin–Ciocalteu reagent, lactic acid, and glucose, while Labkem (Barcelona, Spain) supplied glycerol, and Scharlab Chemie (Barcelona, Spain) provided acetic acid. Ellagic acid (≥95%) and α- and β-punicalagin (≥98%) standards were purchased from Extrasynthese (Genay Cedex, France) and Quimigen (TargetMol; Madrid, Spain), respectively. Ultrapure water was produced using a Millipore system (Millipore, Billerica, MA, USA).

### 4.2. Sample

Peels from pomegranate were purchased from “Empório Grão de Ouro produtos naturais Eirelli” (Guarulhos, SP, Brazil). Then, the raw material was ground using a commercial blender to a particle size between 200 and 500 µm and stored at −20 °C until its analysis.

### 4.3. Synthesis of Natural Deep Eutectic Solvents (NaDESs)

Various NaDESs were chosen based on the literature for extracting phenolic compounds from food matrices, as detailed in Table 1. The selected NaDESs were prepared following the protocol by Hernández-Corroto et al. [46]. The components of NaDESs were mixed at 80 °C for 1 h, using a proportion of 40 wt.% water in NaDESs, until a clear liquid was obtained. This percentage was chosen according to the optimized protocol by Vo et al. [24] for extracting phenolic compounds from black mulberry fruit.

### 4.4. NaDES Characterization

#### 4.4.1. pH

A digital pH meter (model Seven Excellence pH meter S400, Mettler Toledo; Barcelona, Spain) was used to determine the pH values of the NaDESs. Determinations were carried out in duplicate.

#### 4.4.2. Viscosity

The viscosity of a NaDES was determined using a rotational viscosimeter (Visco star-H, J.P. SELECTA, Spain) with an R1 spindle at 200 rpm. The viscosity of a NaDES was evaluated at four different temperatures up to the boiling temperature of water (25, 40, 80, and 100 °C). Determinations were carried out in duplicate, with us expressing the results as mPa·s.

#### 4.4.3. Density

The density of each NaDES was measured in duplicate using a simple gravimetric pycnometer. The pycnometer was weighed before and after filling with a NaDES at different temperatures (25, 40, 80, and 100 °C) and adjusting the volume with water. 

### 4.5. Ultrasound-Assisted Extraction (UAE) and Pressurized Liquid Extraction (PLE)

To select the NaDES to use to obtain high contents of ellagic acid and α- and β-punicalagin from pomegranate peel, UAE was used employing the different NaDESs presented in Table 1. Briefly, 500 mg of sample was mixed with 10 mL of NaDES:water (60:40 wt.%). The mixture was sonicated in an ultrasonic bath (Elmasonic S 10 (H); Elma Schmidbauer GmbH, Singer, Germany) for 50 min at room temperature according to Cyjetko et al.’s [25] protocol. We added 40% water in a NaDES following Cvjetko et al.’s [25] protocol, but also according to previous results (not published) obtained by our research group where different percentages of water in a NaDES were tested for the extraction of phenolic compounds from different batches of pomegranate peels. Then, extracts were centrifuged for 15 min at 5000 rpm. The same UAE procedure was applied using water instead of a NaDES to compare the effect of a NaDES on the recovery of phenolic compounds from pomegranate peel. The supernatants were collected and stored at −20 °C for further analysis.

The total phenolic content and ellagic acid and α- and β-punicalagin contents were used as criteria to select the best NaDES that would be employed in the next step of the work. The selected NaDES was used for solvent extraction in PLE, where the extraction temperature was optimized (40, 80, 120, and 180 °C) according to the contents of the same target compounds previously evaluated. Extractions were performed in a Dionex ASE 200 instrument (Sunnyvale, CA, USA), employing 11 mL extraction cells, which were filled with 1 g sample mixed with 2 g of cleaned sand for 20 min at 1500 psi according to Sánchez-Martínez et al. [29]. Additionally, the same PLE procedure was applied using water instead of a NaDES to compare the effect of a NaDES on the recovery of ellagic acid and α- and β-punicalagin from pomegranate peel. All extractions were carried out in triplicate.

### 4.6. Total Phenolic Content (TPC)

The total phenolic content of the extracts was determined using the Folin–Ciocalteu method, following the protocol by Singleton et al. [47]. A calibration curve with gallic acid (0.05–2 mg/mL) was created to express the results as mg of gallic acid equivalents (GAE) per gram of sample. The determination of the TPC values for each extract was carried out in triplicate.

### 4.7. High-Performance Liquid Chromatography (HPLC-DAD)

The identification and quantification of phenolic compounds from UAE-NaDES, UAE-water, PLE-NaDES, and PLE-water extracts of pomegranate peels were conducted using an Agilent 1100 HPLC system (Agilent Technologies, Palo Alto, CA, USA) equipped with a diode array detector (DAD). The chromatographic separation method was optimized and was achieved using a Gemini NX-C18 column (3 μm, 150 × 2 mm, Phenomenex, Torrance, CA, USA). The mobile phases were water with 0.1% acetic acid (A) and acetonitrile with 0.1% acetic acid (B), utilizing a gradient elution as follows: 3% B (0–15 min); 15% B (15–25 min); 20% B (25–30 min); 25% B (30–35 min); and 90% B (35–40 min), with a post-time of 10 min. The injection volume was 5 µL, the flow rate was 0.250 mL/min, and the column temperature was set at 30 °C. Detection wavelengths were set at 280 and 350. The identification of ellagic acid and α- and β-punicalagin was carried out through a comparison of the retention times and the absorption spectrum of their respective standards. In addition, calibration curves with the respective standards were used to quantify these compounds. The quantification of ellagic acid and α- and β-punicalagin by HPLC-DAD for each extract was performed in triplicate.

### 4.8. Statistical Analysis

Analysis of variance (ANOVA) using Fisher’s exact test was conducted to compare the differences in extraction techniques for total phenolic content, as well as ellagic acid and α- and β-punicalagin contents. This analysis was performed using the statistical program Statgraphics Centurion XVII (Statistical Graphics Corp., Durham, NC, USA).

SIMCA 14.0 software (MSK Data Analytics Solutions, Umetrics, Sweden) was utilized for multivariate statistical analysis. The contents of ellagic acid and α- and β-punicalagin obtained by HPLC-DAD from the optimized UAE-NaDES and PLE-NaDES extractions, as well as their corresponding water extracts from pomegranate peels, were correlated. Additionally, hierarchical clustering analysis (HCA) was performed using Ward’s method and sorted by height. The statistical models were represented as score and loading plots using unsupervised multivariate principal component analysis (PCA).

## 5. Conclusions

This research presents an extraction comparison based on the use of UAE and PLE combined with different NaDESs to recover ellagic acid and α- and β-punicalagin from pomegranate peels. After the NaDES screening process using UAE, the NaDES composed of ChCl:Gly (1:2) was selected as the more efficient solvent to extract phenolic compounds according to their TPC and ellagic acid and α- and β-punicalagin contents as well as their physicochemical characterization (viscosity, density, and pH). This meant that ChCl:Gly allowed a more significant mass transfer into the matrix than the other NaDES formulations under the same extraction conditions (25 °C for 50 min). In addition, UAE-NaDES extraction was compared to UAE-water extraction, with us observing that a NaDES in UAE increased the extraction of target compounds when compared with the use of conventional solvents such as water. In addition, the temperature of PLE-NaDES extraction using ChCl:Gly (1:2) NaDES for 20 min at 1500 psi was optimized and compared with PLE-water extraction to observe the extraction efficiency of the NaDES in PLE. For that, extraction temperatures of 40, 60, 80, and 160 °C were tested, with us obtaining the highest phenolic contents when using 80 °C. Degradation of phenolic compounds at temperatures above 80 °C was observed; thus, a higher temperature than 80 °C is not recommended for the recovery of ellagic acid and α- and β-punicalagin from pomegranate peel. In addition, the effectiveness of PLE in the extraction of phenolic compounds was demonstrated. Higher free ellagic acid and α- and β-punicalagin contents were obtained using PLE-NaDES followed by PLE-water at 80 °C for 20 min at 1500 psi when compared with UAE-NaDES and UAE-water extractions at 25 °C for 50 min. This study revealed that NaDES behavior under a high pressure and temperature increased the recovery of phenolic compounds when compared with ultrasound waves. Therefore, the PLE-NaDES extraction technique employing ChCl:Gly (1:2) as the NaDES could offer a promising tool for extracting ellagic acid and α- and β-punicalagin from pomegranate peels. In that sense, this study provides preliminary results about the use of NaDESs in PLE for the recovery of ellagic acid and α- and β-punicalagin from pomegranate peels, standing as the first step to developing future works based on optimizing the PLE-NaDES extraction conditions using ChCl:Gly (1:2) as the NaDES. This will require investigating the influences of temperature, time, pressure, and % water in the NaDES on the recovery of these compounds.

## Figures and Tables

**Table 1:** Hydrogen bond acceptors (HBAs) and hydrogen bond donors (HBDs) used for NaDES synthesis.

Abbreviation	Component 1 (HBA)	Component 2 (HBD)	Molar Ratio	Reference
ChCl:AA	Choline chloride	Acetic acid	1:1	[23]
ChCl:LA	Choline chloride	Lactic acid	1:1	[8]
ChCl:LA	Choline chloride	Lactic acid	1:2	[8]
ChCl:Gly	Choline chloride	Glycerol	1:2	[8]
ChCl:Glu	Choline chloride	Glucose	1:2	[8]

**Table 2:** Physicochemical characteristics of NaDESs used in this work (pH, density (g/mL), viscosity (mPas·s)).

NaDES	Molar Ratio	pH		Density (g/mL)				Viscosity (mPas·s)			
		25 °C	160 °C	25 °C	40 °C	80 °C	100 °C	25 °C	40 °C	80 °C	100 °C
ChCl:AA	1:1	1.40 ± 0.06 ^c^	1.61 ± 0.07 ^b^	1.10 ± 0.03 ^b^	0.99 ± 0.03 ^b^	0.99 ± 0.02 ^a,b^	1.07 ± 0.04 ^b,c^	6.6 ± 0.2 ^e^	5.6 ± 0.2 ^e^	3.7 ± 0.1 ^d^	3.1 ± 0.2 ^d^
ChCl:LA	1:1	0.80 ± 0.01 ^d^	0.82 ± 0.01 ^c^	1.09 ± 0.02 ^b^	1.027 ± 0.008 ^b^	1.1 ± 0.1 ^a^	1.11 ± 0.02 ^b^	52.6 ± 0.2 ^a^	20.7 ± 0.8 ^a^	15.5 ± 0.7 ^a^	8.7 ± 0.3 ^a^
ChCl:LA	1:2	0.70 ± 0.09 ^d^	0.9 ± 0.1 ^c^	1.11 ± 0.03 ^b^	1.06 ± 0.02 ^b^	1.08 ± 0.03 ^a,b^	1.098 ± 0.008 ^b,c^	8.0 ± 0.3 ^d^	6.7 ± 0.2 ^d^	5.1 ± 0.2 ^c^	3.7 ± 0.1 ^c^
ChCl:Gly	1:2	1.60 ± 0.02 ^b^	1.70 ± 0.04 ^b^	0.984 ± 0.006 ^b^	1.04 ± 0.02 ^b^	1.053 ± 0.007 ^a,b^	1.07 ± 0.02 ^b,c^	10.1 ± 0.3 ^c^	10.5 ± 0.4 ^c^	5.7 ± 0.2 ^c^	3.8 ± 0.1 ^c^
ChCl:Glu	1:2	3.70 ± 0.02 ^a^	3.09 ± 0.02 ^a^	1.20 ± 0.04 ^a^	1.128 ± 0.005 ^a^	1.16 ± 0.02 ^a^	1.23 ± 0.1 ^a^	23.1 ± 0.8 ^b^	15.7 ± 0.6 ^b^	6.7 ± 0.2 ^b^	5.7 ± 0.2 ^b^

^a–e^ Letters indicate statistically significant differences (*p* ≤ 0.05) among NaDESs.

**Table 3:** UAE analytical quantification of ellagic acid and α and β-punicalagin (µg/g sample) by HPLC-DAD from UAE-NaDES extracts.

Compound	Water	ChCl:AA (1:1)	ChCl:LA (1:1)	ChCl:LA (1:2)	ChCl:Gly (1:2)	ChCl:Glu (1:2)
**Ellagic acid**	98 ± 2 ^b^	78 ± 5 ^c^	97 ± 3 ^b^	79 ± 1 ^c^	111.0 ± 0.9 ^a^	62.3 ± 0.9 ^d^
**α-punicalagin**	76 ± 6 ^a^	53 ± 10 ^c^	68 ± 4 ^b^	54 ± 5 ^c^	82.4 ± 0.5 ^a^	36 ± 0.2 ^d^
**β-punicalagin**	55 ± 5 ^e^	96 ± 3 ^c^	111 ± 1 ^a^	89 ± 2 ^d^	105.9 ± 0.7 ^b^	57 ± 2 ^e^

^a–e^ Letters indicate statistically significant differences (*p* ≤ 0.05) among extractions.

**Table 4:** Ellagic acid and α and β-punicalagin contents determined by HPLC-DAD from PLE-NaDES extracts obtained at 40 °C, 80 °C, 120 °C, and 160 °C and aqueous extract obtained at 80 °C.

	PLE-NaDES with ChCl:Gly (1:2)				PLE-Water
**Extraction** **temperature**	**40 °C**	**80 °C**	**120 °C**	**160 °C**	**80 °C**
**Ellagic acid** **(µg/g sample)**	125 ± 2 ^c^	191 ± 36 ^b^	216 ± 3 ^b^	408 ± 7 ^a^	117 ± 6 ^c^
**α-punicalagin** **(µg/g sample)**	79 ± 1 ^b^	93 ± 1 ^a^	82 ± 15 ^a,b^	63 ± 7 ^c^	94 ± 8 ^a^
**β-punicalagin** **(µg/g sample)**	106 ± 1 ^a,b^	114 ± 1 ^a^	92 ± 7 ^b^	97.6 ± 0.4 ^b^	84 ± 5 ^c^

^a–c^ Letters indicate statistically significant differences (*p* ≤ 0.05) among extractions.

## Data Availability

The original contributions presented in this study are included in the article/Supplementary Materials; further inquiries can be directed to the corresponding authors.

## Supplementary Materials

The following supporting information can be downloaded at https://www.mdpi.com/article/10.3390/ijms25189992/s1.
