# Supplementary material for: Advanced Extraction Techniques Combined with Natural Deep Eutectic Solvents for Extracting Phenolic Compounds from Pomegranate (Punica granatum L.) Peels
Source: Int J Mol Sci. 2024 Sep 17;25(18):9992. doi: 10.3390/ijms25189992 (PMC11432524; doi:10.3390/ijms25189992)
Supplement: Supplementary file 1 [file ijms-25-09992-s001.zip › ijms-3213890-supplementary.pdf]

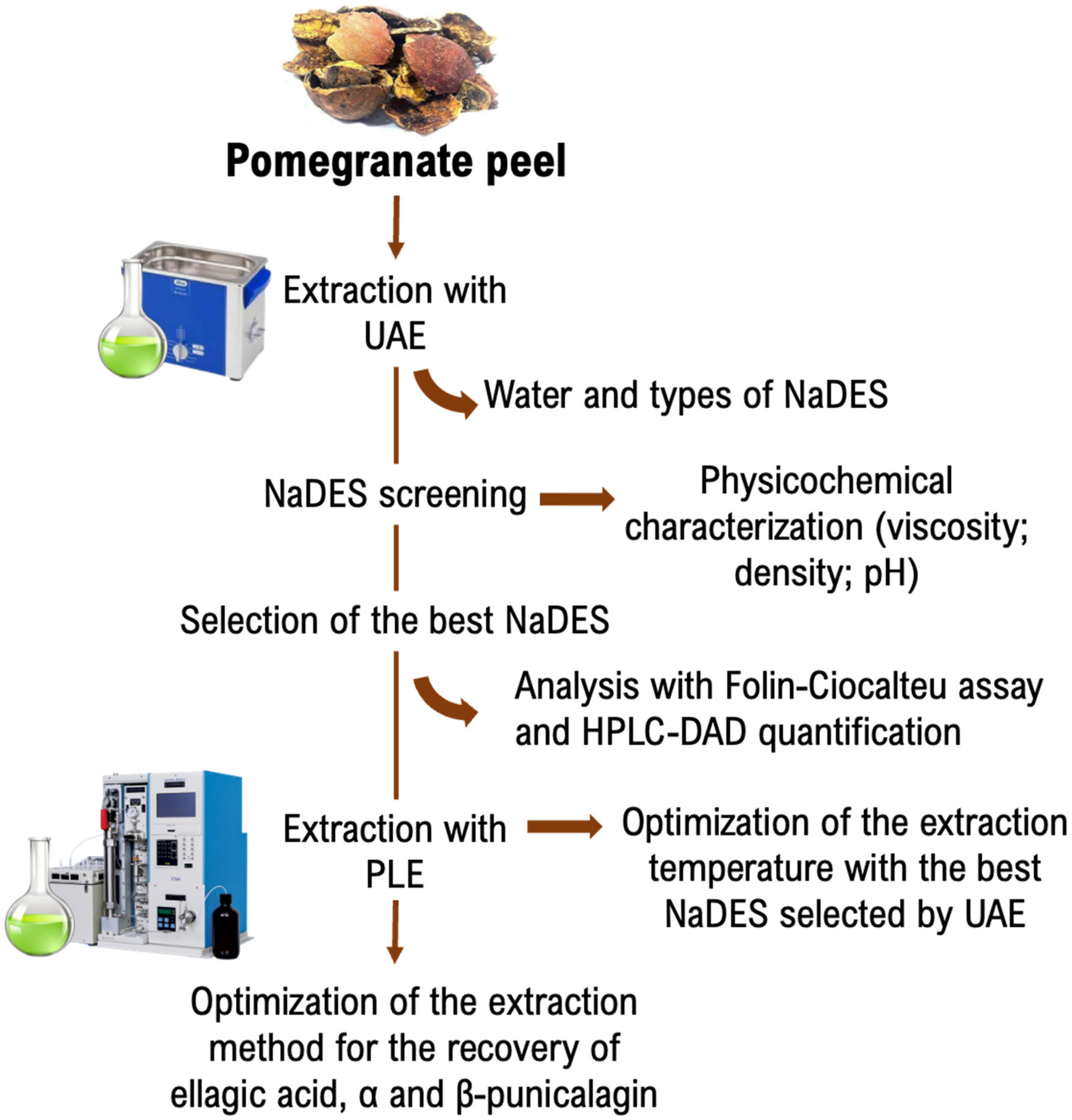

Figure S1. Workflow of the approach followed in the present study for achieving the highest ellagic acid, α- and β-punicalagin content from pomegranate peel using advanced extraction techniques (UAE and PLE) combined with NaDES.
